# Head-to-Head Study of Developmental Neurotoxicity and Resultant Phenotype in Rats: α-Hexabromocyclododecane versus Valproic Acid, a Recognized Model of Reference for Autism Spectrum Disorders

**DOI:** 10.3390/toxics10040180

**Published:** 2022-04-06

**Authors:** Chloé Morel, Armelle Christophe, Katy Maguin-Gaté, Justine Paoli, Jonathan David Turner, Henri Schroeder, Nathalie Grova

**Affiliations:** 1 Calbinotox, EA7488, Université de Lorraine, 54506 Nancy, France; chloe.morel@univ-lorraine.fr (C.M.); armelle.christophe2@gmail.com (A.C.); katy.maguin-gate@univ-lorraine.fr (K.M.-G.); justine.paoli@univ-lorraine.fr (J.P.); henri.schroeder@univ-lorraine.fr (H.S.); 2 Immune Endocrine Epigenetics Research Group, Department of Infection and Immunity, Luxembourg Institute of Health, 29 rue Henri Koch, L-4354 Esch-Sur-Alzette, Luxembourg; jonathan.turner@lih.lu; 3 Inserm U1256, NGERE, Université de Lorraine, 54000 Nancy, France

**Keywords:** α-HBCDD, Autism Spectrum Disorders, early life exposure, brain functionality, neuroglia, synaptic plasticity, neuromotor maturation, noise reaction, rat

## Abstract

Evidence is now growing that exposure to environmental pollutants during the critical early-life period of brain development may contribute to the emergence of Autism Spectrum Disorders (ASD). This study seeks to compare the developmental neurotoxicity of the α-isomer of hexabromocyclododecane (α-HBCDD), a persistent brominated flame retardant, to the valproic acid (VPA) model of ASD in rodents. Pregnant Wistar rats were divided into three groups: control, α-HBCDD (100 ng/kg/day p.o., GD0-PND21) and VPA (600 mg/kg i.p., GD12). Male offspring were tested for their neuromotor development from PND2-21. At PND21, brain functionality was assessed by measuring cytochrome oxidase activity (CO). Modifications in neuroglia and synaptic plasticity were evaluated in the cortex. Similar subtle behavioural changes related to neuromotor maturation and noise reaction were observed in both treated groups. At PND21, a reduction in CO activity was measured in the VPA group only, in specific areas including auditory nuclei, visual cortex, cingulate and frontal cortices. At the same age, α-HBCDD pointed out significant overexpression of cortical markers of synaptic plasticity while both treated groups showed a significant under expression of astrocyte proteins (S100-β and GFAP). Early-life exposure to a low dose of α-HBCDD may trigger neurobehavioural alterations in line with ASD.

## 1. Introduction

Autism Spectrum Disorders (ASD) consist of heterogeneous neurodevelopmental pathologies in their aetiology, manifestations, and potential comorbidities [1]. Their classification and diagnosis are constantly evolving, which led in 2013 to a reassessment and expansion of the diagnostic elements described in the last version of the Diagnostics and Statistics Manual (DSM-5), bringing these disorders together in a broad spectrum under the term ASD [2]. ASD disproportionately affects men, and the male-to-female prevalence of 4:1 is one of the most common findings in the area of ASD [3]. However, this sex difference is most probably due to the difference in pathophysiology, and the lower number of females meeting the diagnostic criteria for ASD [4].

Currently, ASD are recognized as a single group of neurodevelopmental diseases, including autism or autistic disorder, Asperger’s syndrome, invasive unspecified developmental disorder and childhood disintegrative disorder [3]. These pathologies present as aberrant behaviour that appears around the age of 18 months that persists into adulthood. A worrying increase in ASD prevalence has been observed over the last 20 years in industrialized countries, especially in the United States, with 1 in 166 children affected in 2004 compared to 1 in 59 in 2018 [4]. Diagnosis is carried out by observing (1) deficits in communication and social interaction, (2) alteration in the sensoriality and (3) repetitive behaviour associated with restrictive interests [2]. The exact causes of the onset of ASD remain the subject of much investigation since genetic factors account for 20–30% of cases only [2]. In 70% of the cases, the aetiology of ASD is still unknown, and the pathophysiological role of gene x environment interactions, therefore, remains to be elucidated. There is growing evidence that maternal stress exposure affects the neurodevelopment of their offspring [5,6], while recent findings have highlighted that the emergence of ASD could be significantly associated with perinatal exposure to various environmental factors [2]. Three major notable factors can be pointed out: (i) nutritional stress, which includes both nutritional deficiencies (e.g., lower concentration levels of vitamins B6, B9 and B12) [7,8] or excesses [9], (ii) prenatal exposure to pharmacological substances like valproic acid (VPA, 2-propylpentanoic acid), an antiepileptic drug particularly used for drug-resistant epilepsy, and (iii) environmental exposure to chemical stress such as fine particulate matter (PM2.5) [10], polycyclic aromatic hydrocarbons [11,12], heavy metals [13], brominated flame retardants (BFRs) [14,15] and pesticides [16]. Among the environmental chemicals, the role of early exposure to persistent organic pollutants (POPs), especially BFRs, is particularly questioned. In this respect, hexabromocyclododecane (HBCDD) appears as a chemical of notable interest. This BFR is usually used in the thermal insulation of buildings and houses, particularly in Europe. It is composed of three isomers (α, β, γ), with the γ-isomer being the most abundant in the environment, and the α-isomer the most predominant in animal tissues, particularly through dietary and/or atmospheric exposure. α-HBCDD exposure impacts various biological systems such as the thyroid axis [17], the immune system [18] and the liver [19]. These tissues are all highly vulnerable during the perinatal period, and exposure to α-HBCDD will result in long-term consequences, even at low doses [20]. The potential human toxicity of α-HBCDD has been widely established and has resulted in its recent classification as a “Substance of Very High Concern” in Annex XIV of the REACH European Regulation. The lack of information on pregnant women, foetuses and new-born children highlights the necessity to investigate the developmental neurotoxicity of HBCDD.

Previously we investigated the neurodevelopmental toxicity of the α-isomer of HBCDD induced by dietary exposure during pregnancy and lactation in dams at concentrations representative of human exposure. During the first three weeks of life, we observed alterations in the motor maturation of the pups in a dose-dependent manner, regardless of sex [21]. The phenotype, unexpectedly observed in our previous report, was reminiscent of ASD, although the standard ASD tests were not performed at that time. Consequently, further investigations were needed to confirm if early-life α-HBCDD exposure induces a genuine ASD phenotype. Here, we compared α-HBCDD exposure to the valproate model for the study of autism in rodents. Foetal VPA exposure increases the probability of the emergence of ASD in men, which led to the development of the recognized preclinical VPA-exposure model of idiopathic drug-induced ASD [1,22]. The aim of the study was, therefore, to investigate the effects of early-life exposure to α-HBCDD controlled against the recognized VPA model of autism in rats to confirm that α-HBCDD induces a genuine ASD phenotype early in life.

## 2. Materials and Methods

### 2.1. Chemicals

Alpha-Hexabromocyclododecane (α-HBCDD) was purchased from Sigma–Aldrich (St. Quentin Fallavier, France, >98% analytical standard) in powder form. For the mother solution, α-HBCDD was weighed and dissolved in acetone (Acros Organics, Morris Plains, NJ, USA, >99.5% for analysis) at a concentration of 100 µg/mL. The latter was diluted 5 times in acetone to obtain a working solution at 20 µg/mL. Colza oil (Systeme U, Rungis, France), used as a vehicle, was supplemented with 0.1% of the working solution to reach a final concentration of 20 ng/mL. The acetone was allowed to dry at room temperature (±20) until complete evaporation (4 h minimum). The optimal evaporation time was determined as previously described [23]. The dams were weighed daily for the dose of α-HBCDD to be adjusted to the animal’s body weight (b.w). Valproic acid was purchased from Sigma–Aldrich (St. Quentin Fallavier, France, purity 98%) as a sodium salt. The VPA was prepared in 0.9% saline at a concentration of 600 mg/mL. The dosing volume of 1 mL/kg was adjusted to the rat’s body weight on the day of administration.

### 2.2. Animals

Female Wistar rats (*n* = 18, 10 weeks old) were purchased from Envigo (Gannat, France) and pair-housed in open plastic cages. The animals went through a ten-day adjustment period to the conventional controlled environment (a reversed day/night cycle (light turn on at 8.00 p.m., for 12 h), a temperature of 22 ± 3 °C and relative humidity of 50 ± 20%) with access to water and food ad libitum. The females were mated with breeding males, and the reproductive success was evaluated by vaginal smear 12 h later. Females were then removed from the cages and housed individually. This date was considered day 0 of gestation (GD0). The dams were daily exposed to α-HBCDD from GD1 to the day of weaning (postnatal day 21, PND21), whereas VPA was administered i.p. at GD12. The litter size was standardized to 10 pups when necessary to avoid litter effects on pup development.

### 2.3. Doses and Protocol of Exposure

Six pregnant females were randomly assigned to each of the 3 experimental groups: α-HBCDD, VPA and control group. For the α-HBCDD group, the female rats were weighed and administered daily p.o. (gavage) with an appropriate volume of 100 ng/kg/b.w of α-HBCDD in colza oil from the day of fertilization (GD1) to the weaning day (PND21). At GD12, the dams received a single intraperitoneal (i.p.) injection of 0.9% saline. For the VPA group, the female rats were weighed and administered daily with an equivalent oral gavage of colza oil as a vehicle during the same period. At GD12, the dams received a single i.p. injection of 600 mg/kg b.w of VPA in 0.9% saline. The control group received both vehicles only (α-HBCDD free colza oil by gavage and a single dose of 0.9% saline injected i.p.). The male offspring were evaluated for their developmental behaviour between PND2 and PND21. At PND21, the offspring were put to death by decapitation. The brain was collected and extemporarily frozen in a cooled 2-methylbutane at −30 °C bath before storage at −80 °C. The brains were used for histology (*n* = 4) and biochemical exploration (*n* = 3). All animals used in this study were maintained in compliance with the European Union Directive (2010/63/EU) and the local and national ethical guidelines.

### 2.4. Behavioural Testing

#### 2.4.1. Maternal Behaviour and Offspring Monitoring

The dam’s ability to build the nest was recorded on PND9 and expressed as a percentage of success over a 1 h, 2 h and 24 h period (after spreading the sawdust and scattering the pups into the cage), whereas the pup’s retrieving was assessed over a 20 min period on PNDs 4, 7 and 10. The body weight (BW) of the pups was monitored daily from day 2 to 21. At PND21, the anogenital distance (AGD) was measured by means of pictures carried out with the ImageJ software (Bethesda, MD, USA) referred to the diameter of a tube in which the animals were introduced. The ratio of AGD to the cube root of BW (AGD/BW^1/3^) was then calculated.

#### 2.4.2. Neuromotor Development Assessment

All the pups underwent a whole set of behavioural tests related to auditory (auditory reflex assessment between PND11 and PND14) and neuromotor reflex maturation (righting reflex at PND5, grasping reflex at PND6, negative geotaxis at PND9, forelimb grip strength at PND10 and water escape pole climbing (WESPOC) at PND20) as described in Maurice et al. [21]. The tests were performed in a separate room, under red light, between 9 a.m. and 13 p.m. and videotaped. The running order was randomized between the three groups of rats (Control *n* = 19, α-HBCDD *n* = 26, VPA *n* = 19) to prevent a potent effect of circadian variation over the period of observation.

### 2.5. Histochemical Measurement of Cytochrome Oxidase Activity

Histochemical measurements were performed as previously described in Crépeaux et al. [24]. In short, the brains were serially cut on a cryostat in 20 µm-thick coronal sections. One series of sections from each brain was incubated in the dark at 37 °C for 65 min in a solution of 0.04 g cytochrome c from an equine heart, 0.10 g diaminobenzidine (DAB), 8 g sucrose, 0.036 g catalase in 180 mL of 0.1 M phosphate buffer, pH 7. The reaction was then interrupted by rinsing the slides in 0.1 M phosphate buffer, pH 7, with 10% sucrose (*w*/*v*) for 5 min prior to fixing tissue for 30 min. Finally, the slides were washed and dehydrated prior to being coverslipped with Eukitt. All chemicals used were purchased from Sigma–Aldrich (St Quentin Fallavier, France). The analysis of cytochrome oxidase (CO) stained sections was carried out by semi-quantitative densitometry with a BIOCOM 200 computer-assisted image analysis device (Les Ulis, France), in which optical density readings were converted via standards into enzymatic activity values expressed in µmol/min/mg of tissue. Optical density measurements for 27 brain structures, anatomically defined according to the Paxinos and Watson stereotaxic atlas of rat brain (6th edition, 2006) [25], were made in a minimum of 4 adjacent brain sections.

### 2.6. Western Blot

Synaptic plasticity was investigated by measuring synaptophysin, SNAP25 and PSD95 expression, whereas the neuroglia was assessed through the measurement of GFAP, S100-β and Iba1 expression. The cortex was removed by dissection and the proteins were subsequently extracted with RIPA tampon 1X (NaCl 8 g/L, Na_2_HPO_4_ 1.44 g/L, KH_2_PO_4_ 0.24 g/L, 1% Triton, 0.5% DOC, 0.1% SDS) supplemented with phosphatases (1% PMSF, 100 mM Na_3_VO_4_) and a protease inhibitor cocktail (Roche, Boulogne-Billancourt, France). Protein concentration in every sample was assessed by means of the BCA protein assay kit (Thermo Fisher, Illkirch, France). Next, proteins (15 µg) were electrophoresed in 15% sodium dodecyl sulfate-polyacrylamide gel (Bio-Rad, Roanne, France) and subsequently electro-transferred to nitrocellulose membranes (GE Healthcare, Saclay, France). The membranes were treated for 1 h with blocking buffer (200 mM Tris-HCL, pH 7.4, 1.5 M NaCl, 0.1% tween 20, 5% skimmed milk) at room temperature and incubated overnight at 4 °C with the primary antibody: mouse monoclonal anti-S100 antibody (1:2000, Thermo Fisher, MA5-12969), mouse monoclonal anti-GFAP antibody (1:2000, Millipore, MAB360), goat monoclonal anti-Iba1 antibody (1:2000, Novus, NB100-1028), rabbit monoclonal anti-Synaptophysin antibody (1:2000, Novus, NB300-653SS), goat monoclonal anti-Snap25 antibody (1:1000, Santa cruz, SC-20038), anti-PSD95 antibody (1:2000, Novus, NB300-556) or rabbit monoclonal anti-βactin antibody (1:4000, Sigma–Aldrich, A2103). The membranes were washed three times with blocking buffer and incubated with horseradish peroxidase-conjugated anti-mouse (1:5000, R&D Systems, HAF007), anti-rabbit (1:2000, R&D Systems, HAF008) or anti-goat (1:2000, R&D Systems, HAF017) IgG secondary antibody. Immunoreactivity was visualised with an enhanced chemiluminescence kit (GE Healthcare, Buc, France). Images from Western Blot experiments were acquired by using the Image Lab software on a ChemiDoc instrument (Bio-Rad, Roanne, France).

### 2.7. Statistical Analysis

For each group, the normality of distributions (Shapiro–Wilk) as well as the equality of variances (Levene) were assessed. Normally distributed data were analysed by one-way ANOVA, and the main effects (*p* < 0.05) underwent post hoc testing using the Bonferroni procedure. For non-normally distributed data, a non-parametric test was performed using a Kruskal–Wallis test, and then all pairwise multiple comparisons were made with Dunn’s procedure. A Pearson chi-square procedure was used to analyse the maternal behaviour and the auditory reflex assessment. All statistical analyses were carried out using SPSS 16.0 software (SPSS Inc., Chicago, IL, USA).

## 3. Results and Discussion

### 3.1. Gestational Outcomes and Maternal Behaviour

The pregnancy of the females was monitored for any adverse effects of the exposure (Table 1). Exposure to α-HBCDD or VPA did not appear to have an impact on the observed gestational parameters, namely gestation duration, pup number, and sex ratio. A toxicity for pregnant females, resulting in 33.3% mortality (two dams out of the six included in the group), was observed for the VPA group only, which could result in high foetal resorption. Similar acute toxicity was already pointed out by Favre et al., who showed a full foetal resorption rate of 54% in dams administered with the same dose of VPA (600 mg/kg b.w, i.p. G12) [26]. This toxicity seems to be dose-dependent since the resorption rate was reduced by a factor of 2.3 with a 100-fold lower dose [26]. These results were also confirmed by Degroote et al. [27], who observed total resorption of embryos for three out of five females. Nevertheless, the authors did not report dam mortality in their experiment.

At the same time, exposure to α-HBCDD did not appear to exert an effect on the pregnancy parameters. These findings bear out on Saegusa et al. [28], who showed no change in gestation time but differ from several studies that showed an increase in gestation time following exposure to HBCDD [21,29,30]. This discrepancy could be the result of differences in HBCDD-isomer mixtures and exposure doses used. As we previously reported [21], an increase in gestation time was seen following exposure to 22 ng/kg of α-HBCDD but not following exposure to 66 ng/kg. Subtle maternal behaviour changes, in terms of pup retrieving and nest building, were noticed in both α-HBCDD and VPA exposed groups (Table 1). It was observed that mothers exposed to α-HBCDD or VPA retrieved their pups more sporadically than controls at PND10 (100% of pups retrieved in the control group vs. 80% in the α-HBCDD group and 25% in the VPA group, *p* < 0.05). These changes appear to become stronger over time in that there are few to no differences in PND4 between the α-HBCDD- or VPA-exposed groups and the controls. The decreases became noticeable at PND7 (−35% for α-HBCDD and −25% for VPA relative to the controls) and persisted at PND10, where they were more significantly observed for the VPA-exposed group (−20% for α-HBCDD and −75% for VPA compared to controls). On PND9, we observed a delay in nest building in mothers exposed to α-HBCDD compared to the control group at 1 h and 2 h of observation (100% “no building” for the α-HBCDD group vs. 25% for the controls and VPA, *p* < 0.05). All the females ended up building their nests as there was no difference after 24 h (80% full building nest vs. 75% control and 100% VPA, *p* < 0.05). To our knowledge, maternal behaviour has not been previously assessed in the VPA model; however, the later changes observed following exposure to α-HBCDD appear to be similar to that observed in the VPA model, raising the question as to whether a deficit in mother-offspring interaction may be induced in both models.

### 3.2. Effects of α-HBCDD and VPA Exposure on Male Offspring Weight

**Figure 1:**
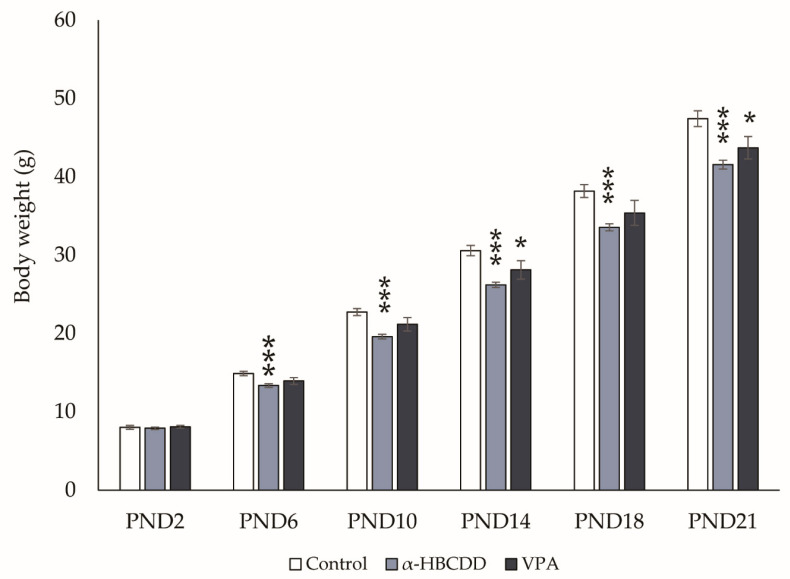
Effects of maternal exposure to α-HBCDD (100 ng/kg/day) or VPA (600 mg/kg) on postnatal body weight of male offspring. Results expressed by mean (Control *n* = 19, α-HBCDD *n* = 26, VPA *n* = 19) ± S.E.M. PND = post-natal day. * *p* < 0.05 vs. Control group; *** *p* < 0.001 vs. Control group.

**Figure 2:**
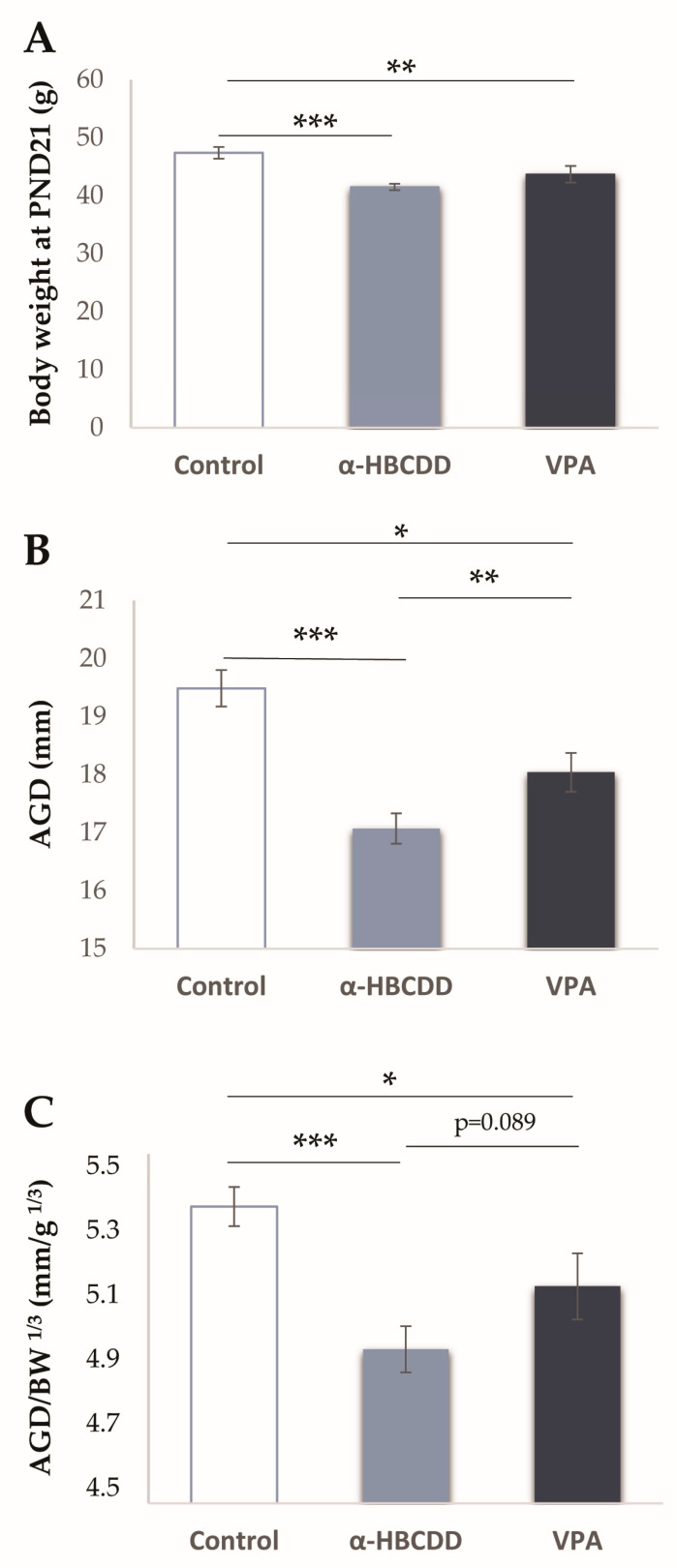
(**A**) Effect of maternal exposure to α-HBCDD (100 ng/kg/day) or VPA (600 mg/kg) on male offspring bodyweight at PND21. (**B**) Effect of maternal exposure to α-HBCDD (100 ng/kg/day) or VPA (600 mg/kg) on anogenital distance (in mm) of male offspring at PND21. (**C**) Effect of maternal exposure to α-HBCDD (100 ng/kg/day) or VPA (600 mg/kg) on anogenital distance/cube root ratio of body weight (AGD/BW^1/3^) of male offspring at PND21. Results expressed by mean (Control *n* = 19, α-HBCDD *n* = 26, VPA *n* = 19) ± S.E.M. PND = post-natal day. * *p* < 0.05 vs. Control group; ** *p* < 0.01 vs. Control group; *** *p* < 0.001 vs. Control group.

Figure 1 and Figure 2A show that all rats were found to gain weight from PND2 to PND21, indicating growth in all groups. However, a difference in growth rate between the groups was noticed, with slower weight gain in both α-HBCDD- and VPA-exposed groups compared to controls. This slowdown was observed at PND6 for the α-HBCDD group (−10%) compared to controls (*p* < 0.001), whereas it appeared only at PND14 for the VPA group (−8%) compared to controls (*p* < 0.05). For the VPA-exposed group, several studies revealed a decrease in body weight of the VPA-exposed animals compared to the controls, which was reproduced in the present results [27,31,32]. Concerning α-HBCDD, Maurice et al. [21] also monitored the weight gain of pups perinatally exposed to α-HBCDD and showed retarded weight gain over the first 4 weeks of life, whereas Miller-Rhodes et al. [29] did not find any differences. These discrepancies may be due to the period of exposure. Miller-Rhodes et al. [29] targeted gestational exposure, whereas both our study and that of Maurice et al. [21] covered gestation and lactation. The fact that no differences in weight gain were observed on the first day of weighing but appeared later may indicate the importance of the lactation period in this weight gain retardation. This difference in weight gain persisted until PND21 in both exposed groups compared to controls (Figure 2A, control: 47.4 ± 0.99 g, α-HBCDD: 41.5 ± 0.56 g and VPA: 43.7 ±1.43 g). This is consistent with other studies conducted on VPA exposure, where a decrease in rat weight was highlighted at weaning age and after [27,32,33]. Lower body weight in the males after exposure to HBCDD was also reported at weaning and later [21,30].

### 3.3. Effects of α-HBCDD and VPA Exposure on Anogenital Distance (AGD)

Due to the potency of α-HBCDD and VPA to act as endocrine disruptors, in addition to the whole body weight, the AGD of the animals was measured at PND21 (Figure 2B). AGD is a recognized marker of endocrine-disrupting effects [34,35]. Decreases in this distance were measured in the groups exposed to α-HBCDD (−12%) or VPA (−7%) compared to the controls (17.07 ± 0.26 mm in the α-HBCDD group with *p* < 0.001 or 18.04 ± 0.34 mm in VPA group with *p* < 0.01 vs. 19.49 ± 0.31 mm in control group, Figure 2B).

A significant difference was also observed between the two treated groups (−5% for α-HBCDD vs. VPA group; *p* < 0.05). To ensure that this difference in AGD was not due to a variation in body weight, the AGD index was calculated according to Gallavan et al. [36], (Figure 2C) as follows: AGD on the body weight cube root (AGD/BW^1/3^ in mm/g^1/3^). The results showed a significant difference between the two treated groups (4.94 ± 0.07 mm/g^1/3^ in the α-HBCDD group with *p* < 0.001 and 5.12 ± 0.09 mm/g^1/3^ in the VPA group with *p* < 0.05) vs. the controls (5.34 ± 0.06 mm/g^1/3^). Based on this AGD marker, these two compounds would therefore be potential endocrine disruptors. For VPA, our results on AGD are not corroborated by other studies [27,37], although AGD is seldom evaluated in the literature. As for α-HBCDD, no differences in the AGD of rats were reported in the literature; nevertheless, the type of mixture, mode of administration and doses of exposure may account for the discrepancy between the results obtained [29,30,38].

### 3.4. Effects of α-HBCDD and VPA Exposure on Motor Development

**Figure 3:**
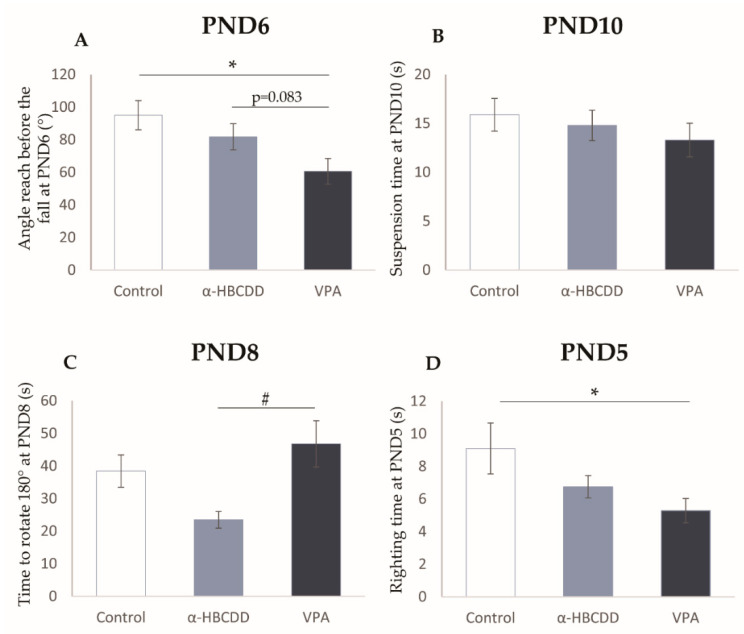
Effects of maternal exposure to α-HBCDD (100 ng/kg/day) or VPA (600 mg/kg) on motor neurobehavioral development of rat pups. (**A**) Angle reach before fall of device in grasping reflex test at PND6. (**B**) Suspension time in suspension test performed at PND10. (**C**) Time to rotate 180° in negative geotaxis test at PND8. (**D**) Righting time in righting reflex test at PND5. Results expressed by mean (Control *n* = 19, α-HBCDD *n* = 26, VPA *n* = 19) ± S.E.M. PND = post-natal day. * *p* < 0.05 vs. Control group; # *p* < 0.05 vs. α-HBCDD group.

A battery of tests was used to assess the neuromotor development of the animals over the first 3 weeks of postnatal life, including the grasping reflex on PND6 (Figure 3A), the forelimb grip strength on PND10 (Figure 3B), the negative geotaxis on PND8 (Figure 3C), the righting reflex on PND5 (Figure 3D), and the WESPOC on PND20 (see the Supplementary Table S1).

Figure 3 displays relative reductions in the grasping reflex (95.1 ± 8.9 degrees in the control group vs. 81.8 ± 8.0 in the α-HBCDD group; or vs. 60.6 ± 7.9 in the VPA group, Figure 3A) and the forelimb grip strength test (15.9 ± 1.7 s in the control group vs. 14.8 ± 1.6 in the α-HBCDD group, or vs. 13.3 ± 1.7 in the VPA group, Figure 3B) in both groups compared to controls, suggesting a short-term reduction in the ability of pups to grasp and maintain their position when suspending.

Regarding the grasping reflex (Figure 3A), a decrease in the average angle reached by the animals before falling from the test device was noted for both the α-HBCDD (−14%) and VPA (−36%, *p* < 0.05) groups with the last one only be significant. Such decreases can be related to the one observed in both groups in the forelimb grip strength test (Figure 3B), albeit not significant. Taken together, these results suggest a delay in this aspect of the development of pups, which is characterised by a lower level of strength necessary in the front legs to grip and hold. Similar changes in forelimb grip strength following exposure to VPA were also previously reported [39]. The same disturbances in the grasping reflex and the forelimb grip strength were reported by Maurice et al. [21] in α-HBCDD-exposed pups of the same age that confirm the ability of this BFR to impair the early motor maturation of the animals, to a less extent compared to VPA.

For the negative geotaxis, the results showed a change in the time required to turn the body around from a head-down position on an inclined plan after exposure to α-HBCDD or VPA (Figure 3C, 38.4 ± 4.9 s in the control group vs. 23.5 ± 2.56 s in the α-HBCDD group; or vs. 46.8 ± 7.12 s in VPA group). There was a non-significant 22% increase in this time point between the control group and the VPA group, as has already been reported in similar literature using a VPA model [31,39]. On the contrary, this time is reduced by −39% in α-HBCDD-exposed rats compared to controls and −50% referred to the VPA-treated animals (*p* < 0.05). Concomitant reductions in the time needed to turn around were observed in the righting-reflex test (Figure 3D, 9.1 ± 1.6 s in the control group vs. 6.8 ± 0.7 s in the α-HBCDD group vs. 5.3 ± 0.7 s in the VPA group, *p* < 0.05) in both groups but with a lesser extend in α-HBCDD compared to VPA. Such changes may therefore indicate an impairment in several aspects of the maturation of the body-balance related systems in the α-HBCDD- as VPA-exposed animals early after birth. Similar changes in motor development, including early swimming performances at PND8 and locomotor/exploratory activities in adolescent and adult animals, have been observed in the same VPA model of ASD in rats [33]. In human studies, motor delay in gross motor skills or fine motor skills or both were also encountered in children with ASD aged 30 months [40]. Finally, in weaning pups (PND21), the results obtained in the WESPOC did not show significant variations in both groups compared to controls (Table S1, Supplemental Data). Nevertheless, a 45% increase in the time used to perform the test in the α-HBCDD exposed group let us suggest a persistent effect. Taken together, all these results highlight that α-HBCDD and VPA trigger motor development in pups in almost similar ways.

### 3.5. Effects of α-HBCDD and VPA Exposure on Auditory Perception

**Figure 4:**
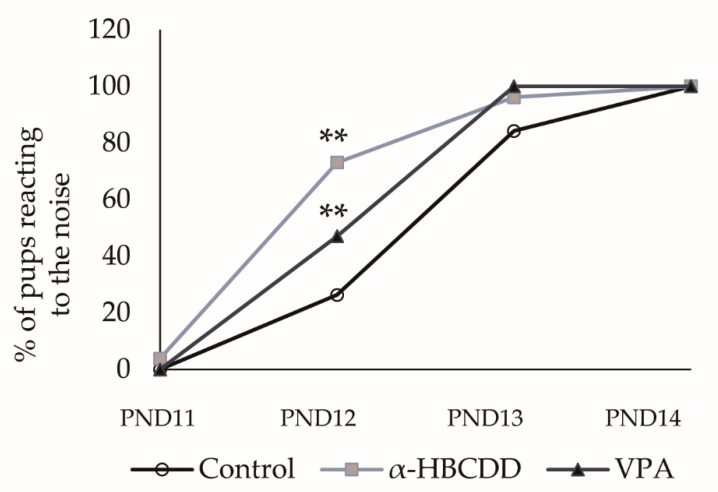
Effects of maternal exposure to α-HBCDD (100 ng/kg/day) or VPA (600 mg/kg) on sensory neurobehavioral development of rat pups with the percentage of pups reacting to noise (startle) in auditory reflex test. Results expressed as percentage. PND = post-natal day. ** *p* < 0.01 vs. Control group.

The final behavioural test carried out introduced a sensory aspect to this study, an essential characteristic of ASD [41]. For this purpose, the auditory startle reflex was tested daily from PND11 to PND14 to evaluate the maturation of the hearing system. At PND11, few rats responded to auditory stimuli (Figure 4, 0% of animals responded in the control and VPA groups vs. 3.85% in the α-HBCDD group) and then started to react at PND12 (26.3% reactions in the control group vs. 73.1% in α-HBCDD group; or vs. 47.1% in VPA group, *p* < 0.01). Over the last two days of testing, almost all the rats responded to the stimulus (PND13: 84.2% reactions in the control group vs. 96.2% in the α-HBCDD group vs. 100% in the VPA group, PND14: 100% reactions for all the groups).

There was an increase in the percentage of rats reacting in all groups over time, thereby confirming a complete auditory development in all groups. At PND12, we observed a significant increase in the percentage of rats responding to noise (+79%) in the VPA group compared to the control group (*p* < 0.01). This difference would indicate a change in sensory maturation with VPA-exposed animals being at a more advanced stage. This group was also the first one to reach 100% of reactions at PND13 compared to only 84.2% for controls. Changes in the hearing reflex development have already been highlighted in this VPA model of ASD with either faster [39] or slower [27] maturation. Hearing impairment is common among children with ASD phenotypes and can substantially vary from one child to another, ranging from hearing loss to hypersensitivity [42]. The variability obtained for the VPA model is therefore consistent with the variability observed in man. The latter was taken into account in the validation of the VPA model as a preclinical model of idiopathic drug-induced ASD [1,22]. Surprisingly, the number of α-HBCDD rats responding to the noise is even greater than in the VPA group at PND12 (+178%, *p* < 0.01). This shows a similar acceleration in sensory maturation after exposure to α-HBCDD, even more acutely than with VPA.

Cytochrome oxidase (CO) activity, considered a marker of neuronal cell function, was then studied in selected brain regions involved in the auditory processing at PND21 (Table 2). Significant changes were observed in four out of the five auditory regions, including the auditory cortex, the medial geniculate nucleus, the inferior colliculus, and the lateral lemniscus. Among the three groups of rats, significant reductions in CO activity were reported only in VPA-exposed animals compared to controls or α-HBCDD-treated rats in these areas (Table 2). No significant variations in metabolic activity were observed in the α-HBCDD-treated group compared to either controls or VPA; however, a similar VPA pattern can be reported with a decrease in CO activity in the inferior colliculus and lateral lemniscus (Table 2).

All the brain areas investigated, interconnected in treating auditory signals from the first synaptic connexions coming from the internal ear to the auditory cortex, exhibited a decrease in CO activity following VPA exposure compared to controls. Such modifications of functional activity in the auditory neural network are correlated with the higher sensitivity to hearing sounds we observed earlier in the same animals. Taken together, these results then suggested a higher sensitivity of the auditory system during development with a potent dysregulation in the perception and brain integration of sounds later in life in both α-HBCDD and VPA treated groups. Thus, the behavioural performances at the adult stage related to hearing sounds, e.g., sound-related conditioning, remain to be explored to confirm the long-lasting changes in this system, especially in conditions of α-HBCDD exposure.

Moreover, differences in CO activity suggest that there are significant changes in activity within the visual cortex in both treated groups compared to controls. Exposure to VPA resulted in a 17% decrease compared to the control group (*p* < 0.05), and exposure to α-HBCDD resulted in an 11% decrease. Alterations in visual sensory maturation have already been shown in this ASD VPA-rat model, as shown by a delayed eye opening [27].

Finally, CO levels were increased in the cingulate cortex after exposure to α-HBCDD (+5%) and decreased by VPA exposure (−17%). A 21% reduction in CO activity was observed between α-HBCDD and VPA (*p* < 0.01). A similar pattern was seen in the frontal cortex with a +15% increase between the control and α-HBCDD group (*p* < 0.01), whereas no significant variation was measured between the control and VPA groups. A −14% drop has been measured in VPA-exposed animals compared to α-HBCDD (*p* < 0.01). Taken together, these results highlight that both types of exposure alter specific areas, including most of the auditory system, visual cortex, cingulate and frontal cortices, known to be highly involved in the onset of ASD.

### 3.6. Neuroglia and Synaptic Plasticity in the Cortex

**Figure 5:**
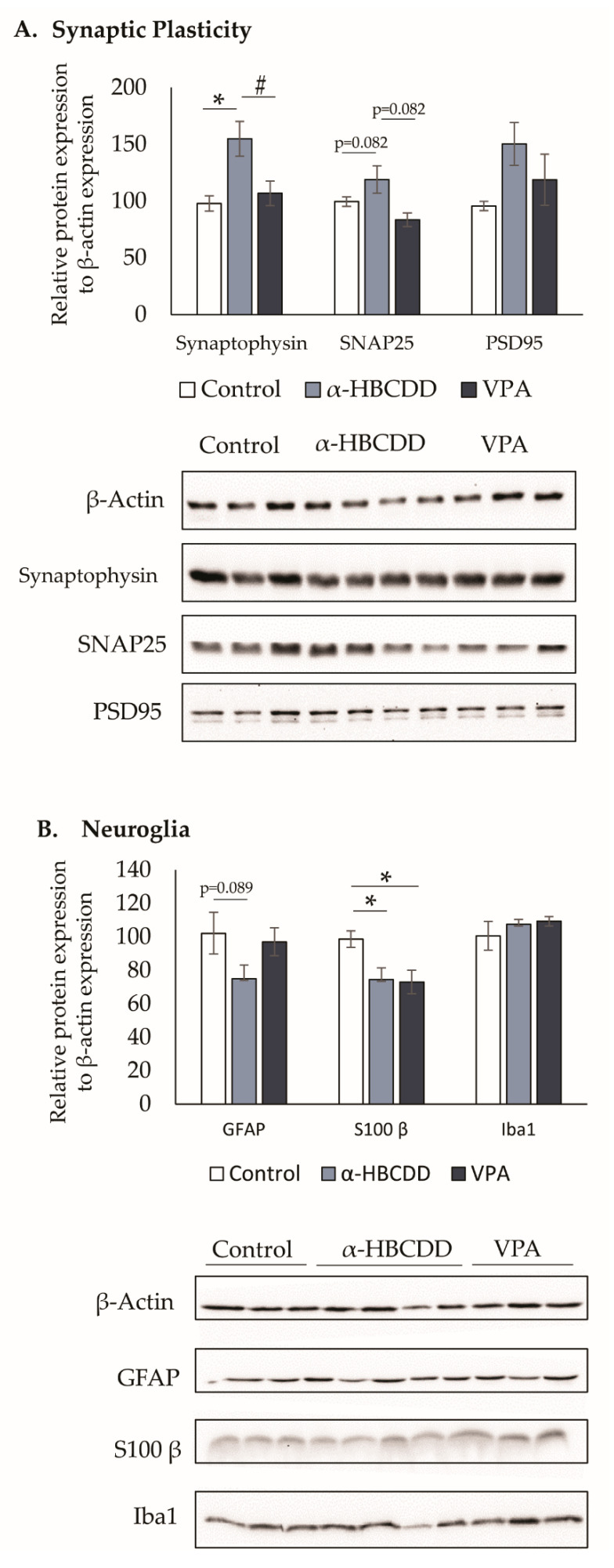
Expression levels of synaptic plasticity (**A**) and glial cell (**B**) markers in offspring cortex following maternal exposure to α-HBCDD (100 ng/kg/day) or VPA (600 mg/kg). The protein expression was determined by western blot on brain samples collected at PND21. Results expressed as mean ± S.E.M with *n* = 3 to 4 per group, 3 to 4 repetitions of measurement. * *p* < 0.05 vs. Control group; # *p* < 0.05 vs. α-HBCDD group.

Neuronal connectivity changes, as well as in glial cells, have been described in different brain regions of the VPA model [43,44,45]. Thus, the expression of synaptic plasticity marker and glial cell proteins was studied by western blot in the cortex of PND21 animals. Synaptic plasticity was assessed via levels of synaptophysin, PSD95 and SNAP25 (Figure 5A). For synaptophysin, we saw an increase between the two exposed groups and the control one of 57% for α-HBCDD and 9% for VPA (97.8 ± 6.7% in the control group vs. 154.9 ± 15.3% in the α-HBCDD group; or vs. 109.9 ± 10.9% in the VPA group, *p* < 0.05). This increase was greater for α-HBCDD and was 31% higher than the VPA group (*p* < 0.05). An increase in this pre-synaptic protein following exposure to VPA was already demonstrated at PND35 in the prefrontal cortex (PFC) of rats [43,45] and more generally in the whole cortex [46]. Here, the α-HBCDD induced increase was even greater than that of VPA, showing a higher number of synapses and/or synaptic vesicles.

For PSD95, even if the results were not significant, there was a trend towards an increase in this post-synaptic marker following exposure to α-HBCDD (+55%) and VPA (+23%) (95.7 ± 4.1% in the control group vs. 150.4 ± 18.7% in the α-HBCDD group; or vs. 118.8 ± 22.5% in the VPA group). This change was again greater with α-HBCDD, with a 21% difference between VPA and α-HBCDD. Previous studies showed an increase in PSD95 in PFC [45] and cortex [46] following exposure to VPA. Since this marker is involved in glutamatergic transmission, these results suggest an increase in the number of excitatory synapses in the cortex following exposure. The last marker of synaptic plasticity investigated in this study was SNAP25, which exerts a role in synaptogenesis as well as in the release of neurotransmitters [47].

Opposite results were obtained according to the exposure to VPA (16% decrease) or to α-HBCDD (19% increase, *p* = 0.082) compared to controls, thus inducing a 30% difference between these two groups (*p* = 0.082). A decrease in SNAP25 in the frontal lobe of the animals in the VPA model has already been reported [48]. In parallel, the results obtained for α-HBCDD are consistent with those observed for tetrabromobisphenol-A (TBBPA; another flame retardant), for which an increase in SNAP25 was observed after in-vitro exposure of primary rat cerebellar granule cell cultures [49]. SNAP25 has been linked to the onset of various psychiatric pathologies (ADHD, schizophrenia or bipolar disorder) [47] and, consequently, is thought to be involved in synaptopathies, including ASD, according to certain authors [50]. Here, we demonstrated early changes in synaptic markers in 21-day-old animals exposed to VPA or α-HBCDD, which may relate to changes in functional activity as previously described in the anterior cortices (Table 2). Alterations in these anterior cortices most probably lead to the development of autistic behaviour later in the life of the rats, since they are heavily involved in social behaviour [51].

Human cohort data suggest that neuronal disturbances may also be associated with glial dysfunctions [52]. In this context, we firstly evaluated the expression of the Iba1 protein, as a marker of microglia, in the cortex (Figure 5B). No differences were observed following exposure to VPA or α-HBCDD in comparison to controls. These results are consistent with those of Traetta et al. [45] and Bronzuoli et al. [44], who also concluded that Iba1 was not affected at PND35 in the VPA model. Subsequently, we examined the glial fibrillary acidic protein (GFAP) and S100-β as markers of astrocytes, known to play a crucial role in brain homeostasis and neuronal and synapse function [53,54]. There was no difference in GFAP between the rats exposed to VPA and controls; in contrast, a trend of 27% lower GFAP levels was seen between the α-HBCDD-exposed group and controls (Figure 5B, *p* = 0.089). Similarly, S100-β was 24% lower after α-HBCDD exposure than the controls and 26% lower in VPA exposed rats than controls (*p* < 0.05). Most of the time, the VPA model is associated with an increase in GFAP [43,45] and the presence of neuroinflammation [55]. Nevertheless, this increase seems to fluctuate over time; in fact, the study of Bronzuoli et al. [44] showed an increase in GFAP at PND35 but not before at PND13 or after at PND90 in the PFC. Given that our sampling age is PND21, potential changes to GFAP by VPA may not yet be apparent. This same study did not reveal any changes in the expression of S100-β, unlike our results showing a decrease in VPA. For α-HBCDD-exposure results, a decrease in both astrocyte markers was observed, which may indicate a decrease in astrocyte cell counts. Madia et al. [56] demonstrated in vitro an increase in the number of apoptotic cells in a culture of human astrocytes following exposure to PBDE-99, another BFR. It is therefore questionable whether the decrease in GFAP and S100-β observed here might be the result of an astrocyte apoptosis induction due to the repeated exposure to α-HBCDD.

## 4. Conclusions

This study aimed to compare the developmental neurotoxicity induced by early-life exposure to α-HBCDD, to that observed in the VPA-exposed ASD reference model in rodents. The effects of α-HBCDD and VPA exposure on neuromotor development and auditory perception were evaluated in males from PND2 to PND21. Similar subtle behavioural changes in neuromotor maturation and noise reaction were observed after exposure to α-HBCDD or VPA. At PND21, clear alterations of neuronal cell function were seen in the VPA group in specific areas, including most of the auditory system, visual cortex, cingulate and frontal cortices. At the same age, α-HBCDD induced a significant overexpression of cortical markers of synaptic plasticity while both treated groups showed a significant under expression of astrocyte proteins (S100-β and GFAP), suggesting a redesign of neuronal networks possibly related to an increase in cell apoptosis induced by both treatments. Altogether, this work clearly demonstrates that early-life exposure to a low dose of α-HBCDD triggers neurobehavioral alterations in line with ASD-like phenotypes.

Although the VPA model has been clearly established in terms of construct and face validity for ASD phenotype in males, the latter is less studied for females. Particular attention should be paid in the near future to sex-specific behavioural records for the characterization and validation of the VPA model. A detailed investigation of the neurotoxicity of α-HBCDD controlled against the recognized valproic acid model of autism in adult rats could subsequently be carried out to confirm that α-HBCDD induces a genuine ASD phenotype in both sexes. The challenge is to now validate these findings later in life and to link the changes reported here with stereotypies, social behaviour disturbances, and other characteristics of ASD phenotypes, as well as to translate this to clinical situations. This can be performed by focusing our interest on fundamental aspects such as neuronal function, synaptic transmission and neuroinflammation, as well as performing complete epigenome sequencing to identify genes that are epigenetically regulated, and the transcriptional consequences, identifying the molecular mechanisms underlying ASD.

## Figures and Tables

**Table 1:** Effects of maternal exposure to α-HBCDD (100 ng/kg/day) or VPA (600 mg/kg) on gestation outcomes, litter size and maternal behaviour. Gestational duration, number of pups (M, F or both) and sex-ratio were analysed using a non-parametric Kruskal–Wallis procedure and scores were analysed using a Chi2 procedure. Gestation time, pup number and sex ratio expressed as median (*n* = four to five per group) and quartiles (in brackets). Maternal behaviour expressed in percentage.

		Control	α–HBCDD	VPA	Factor	Statistical Index	
			(100 ng/kg/day)	(600 mg/kg)		(df)	*p*
***Gestational outcome***							
Number of females included		6	6	6	-	-	-
Reproductive success		5/6 (83.3%)	5/6 (83.3%)	6/6 (100%)	Chi2	1.125 (df = 2)	0.570
Number of surviving females		5/5 (100%)	5/5 (100%)	4/6 (66.7%)	Chi2	3.810 (df = 2)	0.149
Gestation length (days)		22 (22–23)	22 (22–23)	23 (23–23)	K–W	3.900 (df = 2)	0.142
Total number of pups		7 (6–12)	10 (10–12)	10 (10–10)	K–W	0.511 (df = 2)	0.775
Total number of males		4 (4–7)	6 (5–6)	4 (4–6)	K–W	0.473 (df = 2)	0.789
Total number of females		3 (3–4)	4 (4–5)	6 (4–6)	K–W	2.493 (df = 2)	0.287
Sex ratio/litter		1.33 (1.17–2.00)	1.40 (1.33–1.50)	0.83 (0.62–1.67)	K–W	0.748 (df = 2)	0.688
***Nest building (PND9) (*** **%*)***							
1 h	No building	25	100	25	Chi2	n.a. (df = 4)	*p* < 0.05
	Partial building	75	0	75			
	Full building	0	0	0			
2 h	No building	25	100	25	Chi2	n.a. (df = 4)	*p* < 0.05
	Partial building	75	0	75			
	Full building	0	0	0			
24 h	No building	0	0	0	Chi2	n.a. (df = 4)	1.000
	Partial building	25	20	0			
	Full building	75	80	100			
***Pup retrieving (*** **% *of Yes)***							
PND4		50	40	50	Chi2	0.124 (df = 2)	0.940
PND7		75	40	50	Chi2	1.130 (df = 2)	0.568
PND10		100	80	25	Chi2	5.724 (df = 2)	*p* < 0.05

**Table 2:** Effects of maternal exposure to α-HBCDD (100 ng/kg/day) or VPA (600 mg/kg) on brain regional cytochrome oxidase activity (µmol/min/g of tissue) measured at PND21. Results expressed as mean ± S.E.M. of *n* = 4 rat per group; 4 measurements per rat. PND = post-natal day. * *p* < 0.05 vs. Control group; ♦ tendency vs. Control (0.1 < *p* < 0.05); # *p* < 0.05 vs. α-HBCDD group.

Selected Brain Regions	Control	α-HBCDD		VPA		F (2,10)	*p*
		(100 ng/kg/day)		(600 mg/kg)			
**Hearing**							
Auditory Cortex	39.8 ± 1.1	40.0 ± 1.3		32.3 ± 3.1	♦#	4.973	**0.032**
Medial geniculate nucleus	37.0 ± 0.8	38.0 ± 0.9		32.0 ± 1.7	*#	7.856	**0.009**
Inferior colliculus	38.4 ± 0.5	33.1 ± 2.1		29.0 ± 1.7	*	6.960	**0.013**
Superior olive	31.7 ± 0.8	34.4 ± 2.2		20.3 ± 10.4		2.323	0.154
Lateral lemniscus	30.8 ± 1.2	22.9 ± 3.2		19.5 ± 2.5	*	5.467	**0.032**
**Vision**							
Visual Cortex	36.2 ± 1.3	32.4 ± 0.9		30.0 ± 1.5	*	6.541	**0.018**
Superior colliculus	30.6 ± 0.4	33.2 ± 2.2		32.1 ± 0.6		0.731	0.506
**Olfaction**							
Olfactory tubercle	34.9 ± 0.8	35.8 ± 0.5		35.5 ± 0.9		0.346	0.717
Piriform cortex	36.2 ± 4.6	36.0 ± 2.4		35.0 ± 2.0		0.051	0.951
**Mammillary Bodies**							
Lateral core	28.1 ± 1.8	34.3 ± 6.0		27.1 ± 3.6		1.114	0.388
Medial nucleus, median part	29.5 ± 1.9	34.7 ± 2.6		33.0 ± 1.3		1.783	0.247
Medial nucleus, lateral part	31.4 ± 2.2	35.9 ± 2.8		32.3 ± 1.9		0.957	0.436
**Anterior Cortices**							
Cingulate cortex	28.9 ± 0.4	30.4 ± 1.7		23.9 ± 1.0	♦#	6.768	**0.014**
Prelimbic cortex	28.1 ± 0.4	29.9 ± 1.6		25.2 ± 1.5		3.005	0.095
Infralimbic cortex	27.1 ± 0.6	27.9 ± 2.0		26.7 ± 0.6		0.188	0.832
Frontal cortex	31.1 ± 0.9	35.7 ± 0.9	*	30.8 ± 1.0	#	9.135	**0.006**
**Hippocampus**							
CA1	33.1 ± 1.6	33.6 ± 1.8		31.3 ± 2.2		0.407	0.676
CA2	33.1 ± 1.5	36.7 ± 2.3		35.2 ± 2.2		0.759	0.493
CA3	36.3 ± 0.9	39.9 ± 2.4		36.6 ± 2.1		1.029	0.392
Dentate gyrus	33.4 ± 0.4	36.1 ± 2.1		33.9 ± 1.7		0.745	0.499
Entorhinal cortex	31.5 ± 1.8	34.3 ± 1.3		30.1 ± 1.3		2.295	0.151
**Amygdala**							
Central nucleus	33.2 ± 1.7	33.6 ± 2.1		31.8 ± 2.0		0.234	0.796
Medial nucleus antero dorsal	28.8 ± 3.9	30.0 ± 3.2		29.5 ± 1.6		0.041	0.960
Intercalated nuclei	32.0 ± 1.7	32.6 ± 1.8		31.9 ± 2.3		0.036	0.964
Basolateral nucleus	34.0 ± 2.6	34.2 ± 2.3		30.4 ± 1.3		0.990	0.409
Basomedial nucleus	28.6 ± 3.9	30.5 ± 3.0		27.9 ± 2.1		0.213	0.813
**Cerebellum**							
White matter	19.2 ± 0.1	12.7 ± 2.8		18.7 ± 2.1		2.911	0.101

## Data Availability

Not applicable.

## Supplementary Materials

The following supporting information can be downloaded at: https://www.mdpi.com/article/10.3390/toxics10040180/s1, Table S1: Effects of maternal exposure to α-HBCDD (100ng/kg/day) or VPA (600 mg/kg) in the water escape pole climbing (WESPOC) test at PND20.
