# Supplementary material for: Head-to-Head Study of Developmental Neurotoxicity and Resultant Phenotype in Rats: α-Hexabromocyclododecane versus Valproic Acid, a Recognized Model of Reference for Autism Spectrum Disorders
Source: Toxics. 2022 Apr 6;10(4):180. doi: 10.3390/toxics10040180 (PMC9029525; doi:10.3390/toxics10040180)
Supplement: Supplementary file 1 [file toxics-10-00180-s001.zip › toxics-1665217-supplementary.pdf]

toxics

MDPI

Supplementary Materials: Head-to-Head Study of Developmental Neurotoxicity and Resultant Phenotype in Rats: α-Hexabromocyclododecane *versus* Valproic Acid, a Recognized Model of Reference for Autism Spectrum Disorders

Chloé Morel, Armelle Christophe, Katy Maguin Gaté, Justine Paoli, Jonathan D. Turner, Henri Schroeder and Nathalie Grova

Table S1. Effects of maternal exposure to α-HBCDD (100ng/kg/day) or VPA (600mg/kg) in the water escape pole climbing (WESPOC) test at PND20. Results expressed by mean ± S.E.M. PND = post-natal day.

	**Control**	**α-HBCDD (100 ng/kg/day)**	**VPA (600 mg/kg)**	**F (2,58)**	**p**
Time to perform the test (s)	28.5 ± 4.5	40.12 ± 10.5	25.9 ± 3.2	0.532	0.766
Successful pups (%)	100	96	100		
**Time to perform the different phases of the test (s)**					
Swimming into the pool	9.7 ± 1.1	13.5 ± 5.3	8.3 ± 1.3	0.481	0.621
Climbing the metal rod	22.9 ± 5.1	25.8 ± 8.2	17.6 ± 2.5	0.38	0.685
Landing the platform	198.1 ± 12.4	192.8 ± 12.4	213.1 ± 3.3	1.908	0.157
